# Circadian rhythms of migraine attacks in episodic and chronic patients: a cross sectional study in a headache center population

**DOI:** 10.1186/s12883-018-1098-0

**Published:** 2018-07-02

**Authors:** Marina de Tommaso, Marianna Delussi

**Affiliations:** Applied Neurophysiology and Pain Unit, Basic Medical Science, Neuroscience and Sensory System-SMBNOS-Department, Policlinico General Hospital, Bari Aldo Moro University, Giovanni XXIII Building, Via Amendola 207 A, 70124 Bari, Italy

**Keywords:** Migraine, Circadian rhythm, Central sensitization, Clinical correlation

## Abstract

**Background:**

Migraine is considered a disease with diurnal and 24 h pattern, though the existence of a prevalent circadian rhythm associated to migraine frequency and severity is still not clear. This observational cross-sectional study aimed to:

1. Assess the circadian rhythm of migraine attacks onset in a large patients’ population selected in a headache center and including episodic and chronic migraine 2. Analyze the principal characteristic of the different onset time groups 3. Verify if migraine features, particularly those associated to chronic and disabling migraine, could be discriminant factors for time of onset group.

**Methods:**

We selected 786 consecutive migraine outpatients, who correctly completed the headache diaries for 3 consecutive months and who fulfilled the diagnosis of migraine without aura-MO, migraine with typical aura alone or associated to migraine without aura - MO/MA and chronic migraine – CM. For the time of headache onset, we considered four time slots, from 6 to 12 am (morning), from 1 to 6 pm (afternoon), from 7 to 11 pm (evening), from 12 pm to 5 am (night), and an additional category named “any time”. Each time slot included the 60 min preceding the next one (e.g. an onset at 12.30 am was included in 6–12 am time slot). We evaluated in all patients the pericranial tenderness, anxiety and depression tracts, headache-related disability, sleep features, quality of life, allodynia and fatigue.

**Results:**

We scored a total of 16,578 attacks, distributed in the entire day. The most of patients, including CM, satisfied the criteria for the “any time” onset. Night onset was significantly less represented in the MA/MO group. Patients with prevalent night onset were significantly older, with longer migraine history and shorter sleep duration. Age and illness duration were the variables discriminating the different onset time groups.

**Conclusions:**

The most of migraine patients do not report a specific circadian profile of attacks occurrence. Frequent migraine, severe disability, psychopathological tracts as well as central sensitization signs, do not match with a specific circadian rhythm of attacks onset. Night onset migraine seems to be an age related feature, emerging in the course of the disease.

**Electronic supplementary material:**

The online version of this article (10.1186/s12883-018-1098-0) contains supplementary material, which is available to authorized users.

## Background

Migraine is considered a disease with diurnal and 24 h pattern [1]. The periodicity of migraine was frequently demonstrated in observational studies [2–5]. Fox et al. [2], reported prevalent morning onset between 6 to 9 am, though a high number of acute headache episodes occurred along all day, with some nocturnal cases [2]. Alstadhaug et al. [3, 4] described a wide distribution of time of occurrence [4]. A recent study based on general population, assessed the circadian periodicity of migraine patients [5]. In this large database, attacks of migraine frequently occurred early in the morning, characterizing patients with early chronotype. A recent study described the circadian rhythm of migraine patients, who used a on-smartphone headache diary. This could be a reliable and handful method to assess migraine features [6]. In this study, both migraine and not migraine headache occurred preferentially in the early morning, though many acute headache episodes occurred during other hours, including night [6]. These studies spotted a prevalent morning onset of migraine, but the correlation between circadian rhythm and clinical phenotype, as migraine frequency and disability, might be worthy of further evaluation. No study included chronic migraine, possibly because the assessment of headache onset is difficult to spot in patients with persistent and even continuous pain. The recent classifications [7, 8] request at least 8 typical migraine attacks in 1 month, to confirm the diagnosis of Chronic Migraine. If patients are requested to distinguish acute migraine from continuous headache, they could also indicate migraine onset time. Another unclear point is the possible association between circadian rhythm and symptoms of central sensitization as allodynia and pericranial tenderness [9, 10].

This observational cross-sectional study aimed to:

1. Assess the circadian rhythm of migraine attacks onset in a large patients’ population collected in our headache-centre and including episodic and chronic migraine.
2. Analyse the principal characteristic of the different onset time groups.
3. Verify if migraine features could be discriminant factors for time of onset group, focusing in particular those associated to chronic and disabling migraine, as pericranial tenderness, allodynia, sleep time, anxiety and depression tracts.

## Methods

### Study population

Study population included outpatients who came for the first time to the Bari Policlinico General Hospital - Applied Neurophysiology and Pain Unit, in the time between January 2015 – January 2017. Upon the first access to the booking desk, the hospital staff gave patients the headache diary. All patients were invited to fill the headache diary for 3 months, and to present it at their next visit date. We decided to include only patients at their first access to our Unit, because one exclusion criteria was the use of preventive treatment for migraine, which we generally used to suggest during the first visit.

### Data collection

The hospital staff requested the patients to complete the hourly chart of headache, reporting: the time of onset of each migraine attack, the quality (throbbing, oppressive, penetrating) and location of headache, the intensity of headache in a scale from 1 (slight) to 3 (intense), the presence of prodromal and aura symptoms, the presence of nausea, vomiting, phono/photo-phobia, and drugs used for single attacks. A sample of original hourly chart and diary of headache can be seen in the supplementary section (Additional file 1: Figure S1, Additional file 2: Figure S2). They also requested to fill the questionnaire of allodynia [11, 12], adapted in the Italian version, for each migraine attack (Additional file 1: Figure S1) [13, 14]. Inclusion criteria were: 1) the diagnosis of migraine without aura-MO (code 1.1), migraine with typical aura-MA (code 1.2.1), migraine with typical aura and migraine without aura MO/MA (code 1.2.1 plus 1.1) and chronic migraine – CM (code 1.3) [7]; 2) age between 18 and 75; 3) at least two migraine attacks in the 3 months; 4) the correct compilation of the hourly chart of headache, with at least the presence of aura symptoms, and the allodynia questionnaires. Exclusion criteria were: general medical and/or other neurological or psychiatric diseases, the use of central nervous system-active drugs or preventive treatment for primary headache, the diagnosis of “probable” migraine, according to classification criteria [7].

Many patients did not report in a complete and precise way the information required, like intensity of headache, vegetative symptoms and use of symptomatic drugs. We did not include these data in the final analysis. For all patients and specially CM, we used these data to confirm the diagnosis, according to the current classification [7, 8].

### Clinical assessment

Two neurologists with clinical experience in headache, put the diagnosis of migraine based on headache characteristics and frequency, in the 3 months preceding the first visit, according with the International Headache Society criteria. The medical staff revised the diagnosis retrospectively, considering the most recent criteria [8]. The composition of study population remained exactly the same, as the new diagnostic criteria for migraine did not change from the previous version [7]. During the first visit, patients underwent the clinical assessment that we described in previous studies [13, 15]. First, the neurologists checked the frequency of migraine, and the averaged number of days with headache/month during the last 3 months, then considered headache intensity and the vegetative and or aura symptoms, to confirm migraine diagnosis. They confirmed the presence of acute allodynia symptoms from the allodynia questionnaire [11, 12]. We classified a patient as allodynic if he/she reported at least one symptom included in the questionnaire in over the 50% of the migraine episodes.

For the time of headache onset, we considered four time slots, from 6.00 to 12.00 am (morning), from 1.00 to 6.00 pm (afternoon), from 7.00 to 11.00 pm (evening), from 12.00 pm to 5.00 am (night), and an additional category named “any time”. Each time slot included the 60 min preceding the next one (e.g. an onset at 12.30 am was included in 6–12 am time slot). We thus included each patient in a specific time of onset category, based on the 50% of his/her migraine attacks occurrence. Alternatively, if migraine attacks happened in different day times, the patient was included into the “any time” category.

We evaluated in all patients the total tenderness score (TTS), following the procedure described by Langermark and Olesen [16]. We also evaluated anxiety and depression tracts using the depression –self rating depression scale (SDS)- and anxiety -self-rating anxiety scale (SAS)- scales, which we applied in large primary headaches groups [13, 14]. They are reliable tools to detect these symptoms in a general non-psychiatric patient population [17, 18]. According to previous studies [13, 14] we applied the Italian version of the MIDAS score to all type of headaches [19], to quantify headache-related disability.

All patients reported on interview their sleep features, according to the Medical Outcomes Study (MOS) [20], on a scale we previously applied in large headache populations [13, 14]. For the present analysis, we considered the sleep quantity score (MOS2); lower scores indicated worse sleep problems, referring to hours of sleep for night in the last week.

We evaluated the patients’ quality of life using The Short-form 36 Questionnaire (SF-36), with the computation of Physical and Mental Health score (PH and MH) [21].

### Ethical approval

The Ethical Committee of Bari Policlinico General Hospital approved the study. All patients signed an informed consent for the inclusion of their data in the study.

### Statistical analysis

Preliminary descriptive analysis of the sample. We summarized the quantitative continuous variables (age, duration, frequency, SAS, SDS, TTS, MOS2, PH and MH sub scores of SF36) with mean and standard deviation, and where necessary, with median and also the 25th and 75th percentile (MIDAS). We summarized the categorical variables (sex, presence or absence of fatigue and allodynia) with absolute frequencies and percentages.

The statistical analysis included the chi-square to test for the categorical variables, the variance analysis (ANOVA) and the Kruskal Wallis’ non-parametric test for the quantitative continuous variables. We used the Bonferroni post-hoc test. The statistical significance level was fixed at alpha < 0.05.

To satisfy the aim 1) we applied the chi square test to verify the distribution of migraine patients into the different time of onset categories. For aim 2) we thus performed a MANOVA analysis, with a complete factorial Type III model, comparing continuous variables (age, duration, frequency, SAS, SDS, TTS, MOS2, PH and MH sub scores of SF36) among onset time categories, followed by a post-hoc Bonferroni test. Using the chi square test, we evaluated the distribution of patients with or without allodynia and fatigue among the different onset times.

To satisfy the aim 3), we applied a step way discriminant analysis with Wilks lambda model (*p* < 0.05 to entry, *p* 0, 10 to exclude), to establish if time of onset category could predict clinical features, as age, duration and frequency of migraine, anxiety and depression scores, quality of life, pericranial tenderness, allodynia, sleep time.

We used the Statistical Package for Social Science (SPSS) version 24 software.

## Results

### Demographic and clinical characteristics

**Fig. 1:**
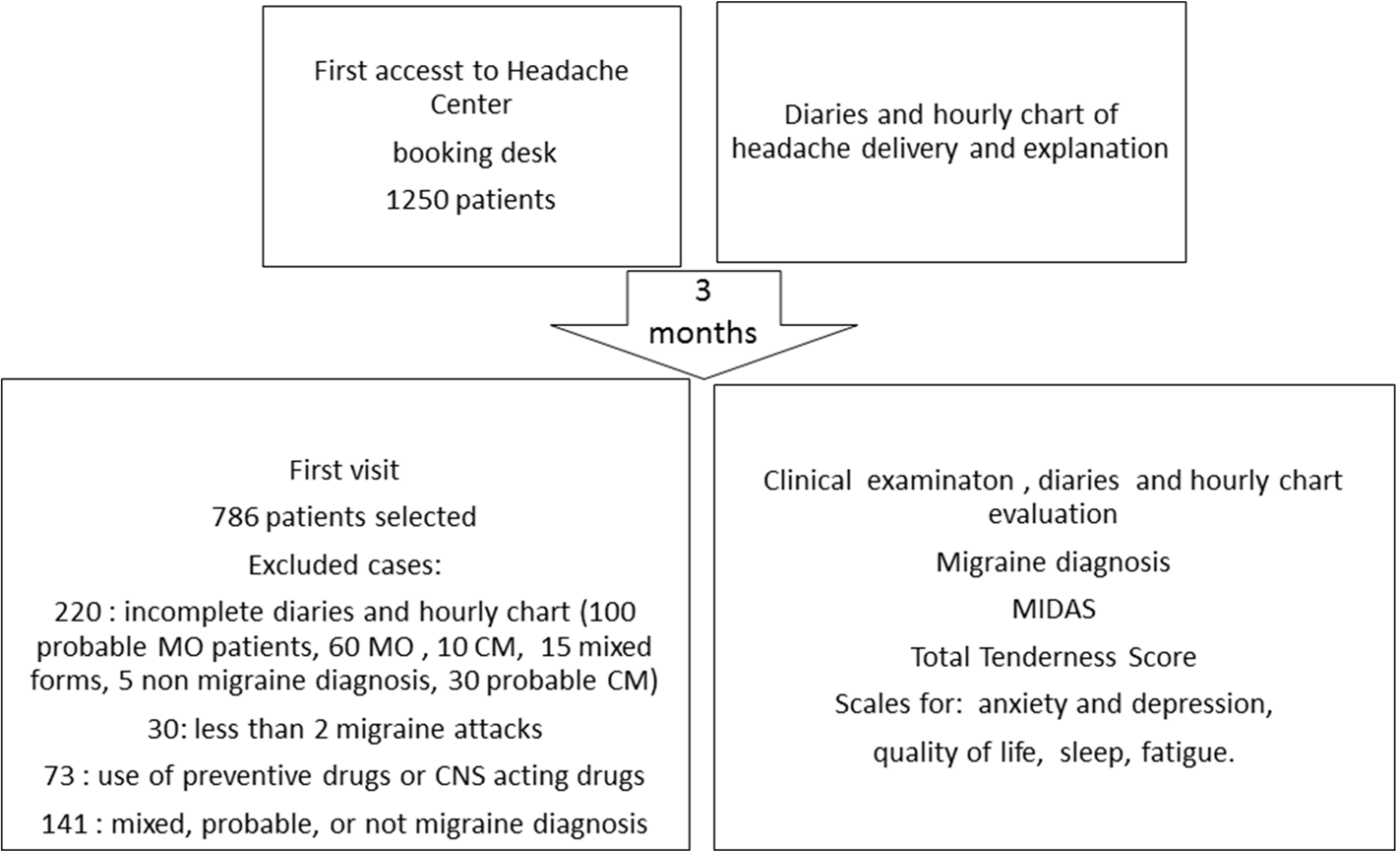
Flow chart reporting study design and patients selected

Among a total of 1250 patients who came at the time of visit booking, we considered data from 786 cases (Fig. 1). Two hundred and twenty patients did not correctly complete the diaries and the hourly chart, 30 reported less than 2 migraine attacks, 73 were under CNS active drugs, 141 patients did not fulfil the diagnostic criteria, including those with mixed diagnosis, as chronic patients with associated Medication Overuse Headache (Fig. 1). In fact, the initial number of CM patients was 290, including 150 patients with an adjunctive diagnosis of Medication Overuse Headache (cod 8.2) [7]. After 3 months of drugs withdrawal and detoxification, 94 of them reverted into episodic migraine, so we excluded their data from the final analysis, which included 196 CM patients.

Considering that few migraine patients presented exclusively with migraine with aura episodes (10 patients), we merged MA and MO/MA patients in a unique group.

The 786 patients enrolled for the study, were all born and resided in Southern Italy. They reported in total 16,578 attacks on their diaries. Females prevailed in all group. CM patients were older than the other patients were. Duration of illness was also longer in CM patients, as compared to other groups (Table 1).

**Table 1:** Demographic and clinic characteristics of migraine patients

	MO		CM		MO + MA		
	N	%	n	%	N	%	
	538	68.4	196	24.9	52	6.6	
Males	123	22.9	23	11.7	10	20	Chi square 0.011
Females	415	77.1	173	88.3	42	80	
	Mean	SD	mean	SD	Mean	SD	One way Anova
Age (years)	37.41*	12.457	42.49*^°	14.656	34.62^	13.45	0.0001
Duration (years)	15.03*	12.87	18.05*	12.599	14.38	11.38	0.008
Frequencies (day/headache/month)	7.64*	6.43	23.35*migraine days 10.15	14.83 3.43	7.7^	6.9	0.0001
SAS	34.06*	7.82	39.12*^°	10.02	32.8^	8.66	0.0001
SDS	32.7*	8.4	38.04*^°	11.018	31.4^	8.45	0.001
TTS	3.86*	4.44	7.02*^	6.037	5.45	5.36	0.0001
MOS2	6.54*	1.35	6.122*^°	1.6284	6.79^	1.13	0.001
PH	36.23	8.71	34.43	8.846	36.94	9.86	n.s.
MH	39.55	7.85	36.56°^	7.044	41.45^	9.99	0.0001
	Median	25°-75°	Median	25°-75°	Median	25°-75°	Kruskal Wallis
MIDAS	6.7*	1.3–16	23.3*^°	8.5–44	3.7^	3–8.5	0.0001

The *p* values are reported for the statistical tests applied. Results of post-hoc Bonferroni test: *CM vs MO; ^CM vs MA; °CM vs MO + MA *p* < 0.05

*MO* Migraine without aura

*CM* Chronic Migraine

MO + MA Migraine without aura + migraine with aura

*SAS* Self Administered Zung Anxiety Scale

*SDS* Self Administered Zung Depression Scale

*TTS* Total Tenderness Score

*PH* SF36 Physical Health score

*MH* SF 36 Mental Health score

Chronic migraine patients presented with higher anxiety and depression scores, as compared to the other groups. Pericranial tenderness score was also higher in CM patients, as compared to episodic migraine without aura and migraine with aura. Mental quality of life score was also lower in chronic patients, compared to migraine with aura patients and mixed forms. The hours of sleep were also fewer in CM patients, as compared to the remainder migraine patients. In addition, the MIDAS scores, which were summarized as median and percentiles, indicated a more severe disability in CM patients (Table 1).

Fatigue characterized a minority of episodic migraine patients, and around the half of chronic migraine subjects. The presence of allodynia prevailed in all migraine groups, but it was more severe in CM patients compared to episodic migraine without aura and migraine with aura groups (Additional file 3: Table S1).

### Time of onset

**Fig. 2:**
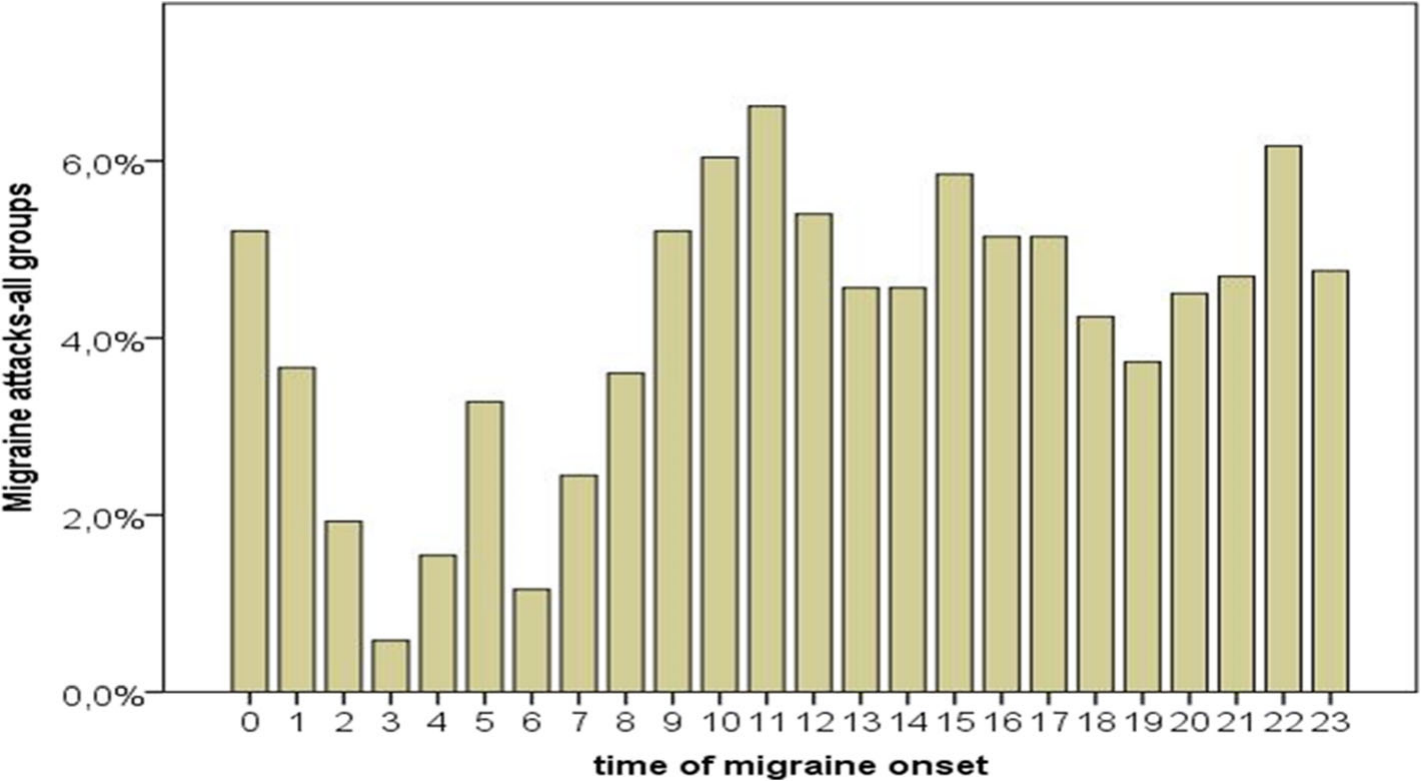
Circadian rhythm of migraine attacks in the 786 patients. The 16.578 attacks were divided among the 24 h. The distribution was not uniform, for a reduction of the attacks in the night hours. (Bootstrap, Tukey estimator 4.49, distorsion − 0.03)

The distribution of the single migraine attacks covered the entire day, with two peaks of migraine frequency at 10 am and 10 pm. A consistent number of attacks occurred in the night, with a peak around middle night (Fig. 2). However, the number of attacks during night were less than in the morning, afternoon and evening hours, so the distribution was not uniform (Fig. 2).

Most of patients satisfied the criteria for the “any time” category, as they did not report a constant time of migraine onset across their attacks (Table 2). The distribution into the different time of onset slots was similar in CM compared to episodic migraine groups. Few patients with aura symptoms reported a prevalent night onset. The comparison of distribution into the different onset time slots between patients presenting or not with aura symptoms, was significant for a lower percentage of night onset patients in the MO/MA group (MO/MA 9%, CM and MO: 25, 7% chi square 16, 3 *p* 0.003).

**Table 2:** The distribution of patients in the five categories of migraine time of onset

			MO	MO + MA	CM	total
Time of migraine onset	MORNING	n	48	9	23	80
		%	8.9%	17.3%	11.9%	10.2%
	AFTERNOON	n	37	5	13	55
		%	6.9%	9.6%	6.7%	7.0%
	EVENING	n	6	1	3	10
		%	1.1%	1.9%	1.5%	1.3%
	NIGHT	n	136	5	51	192
		%	25.2%	9.6%	26.3%	24.4%
	ANY TIME	n	312	32	104	449
		%	57.9%	61.5%	53.6%	57.1%

Subsets of diagnosis were not different from each other at level 0, 05. The “any time” group included the most of patients. Chi Square test: 24, 64; DF 8; *p* 0.02

### Clinical characteristics of time of onset groups

Patients with presence of fatigue and allodynia symptoms were similarly distributed among different onset time slots (Additional file 3: Table S2 and Additional file 3: Table S3).

**Fig. 3:**
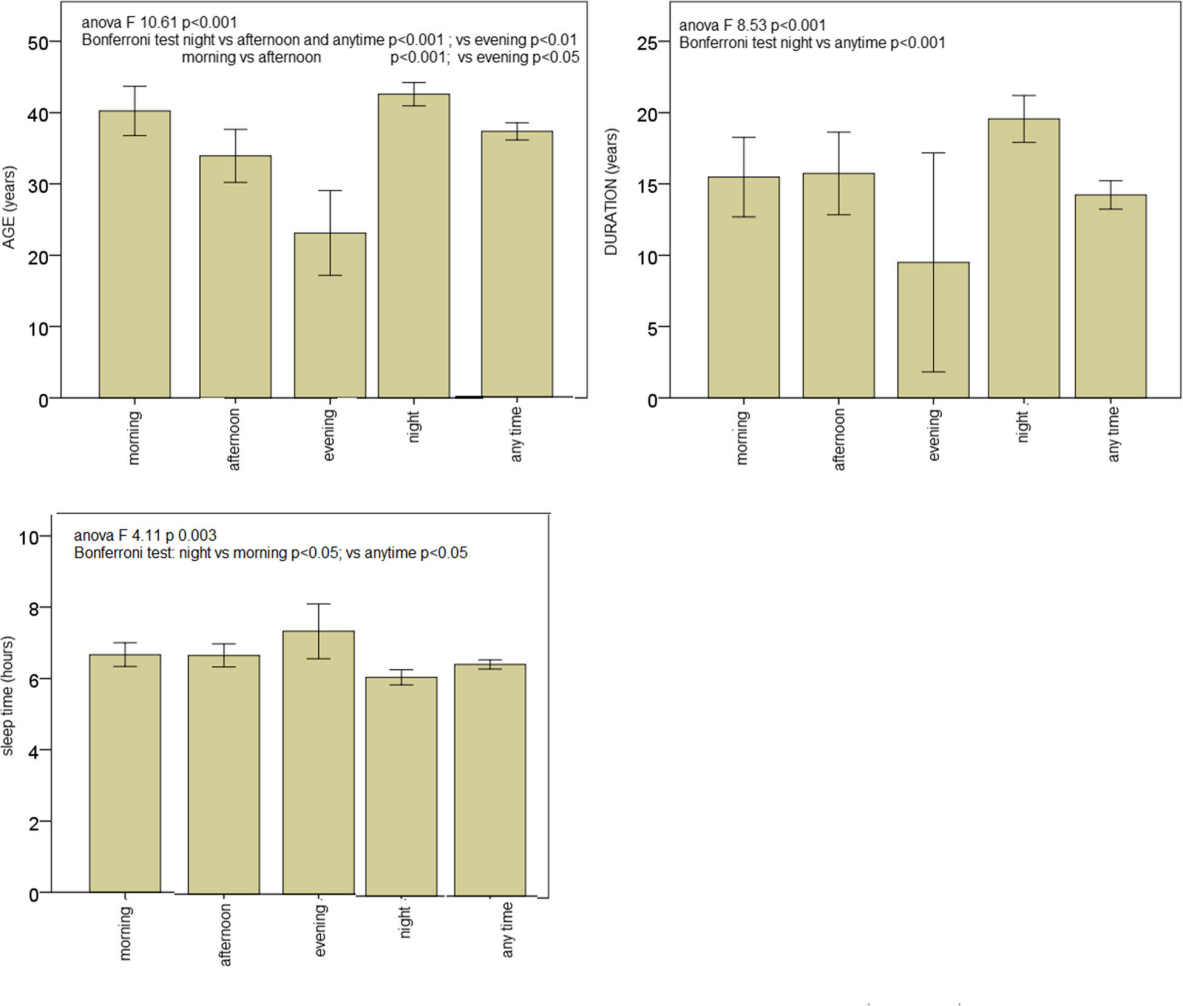
Mean and 95% Confidence Intervals of variables which resulted significantly different in the comparison among the different time onset groups. The ANOVA and post hoc Bonferroni test for single variables are reported

The MANOVA analysis, indicated that the onset time groups, were significant different for the included variables (Roy Square DF 10 *p* < 0.0001). Age, duration of illness and sleep time, showed significant differences among onset time groups (Fig. 3). Patients with prevalent night onset, were older than the other groups, excluding morning onset patients, and reported a longer migraine duration as compared to the “anytime” group. The night onset patients had a shorter sleep, as compared to morning and anytime onsets groups. Patients with morning onset were also older than those with evening and afternoon onset (Fig. 3). The other variables, including allodynia, frequency of headache and quality of life scores, were similar among onset time groups.

The MIDAS scores were also similar among the different onset time slots (chi square 3.48 *p* 0.54).

### Discriminant analysis

The step way discriminant analysis, indicated age and illness duration as discriminant variables (Table 3; Additional file 3: Table S4). The discriminant matrix correctly classified into time of onset groups the 27.6% of patients. The night onset group had the best accuracy, with 46% of correct classification.

**Table 3:** Results of step way discriminant analysis for the onset time groups

Included variables^.b.c^									
step	Included	Wilks Lambda							
		Statistic	df1	df2	df3	F			
						Statistic	df1	df2	Sig.
1	AGE	.942	1	4	711	10.962	4	711.000	.000
2	DURATION	.918	2	4	711	7.749	8	1420.000	.000

The variables initially introduced were age, duration, frequency of migraine, sleep duration (MOS score), anxiety and depression scores (SAS and SDS), SF36 quality of life subscores (PH and MH), Total tenderness score, allodynia total score

a. maximal step number 20

b. inclusion significance for F 0.05

c. exclusion significance for F 0.10

## Discussion

The main result of this observational study in a population selected at headache centre, indicates that the most of migraine patients, including chronic migraine, do not report a constant circadian rhythm of their attacks. In addition, we did not find an association between a specific circadian recurrence of attack and clinical features. Patients with prevalent night onset were older and reported a longer history of migraine. The following paragraphs deal with the detailed discussion of the results.

Concerning the general clinical characteristics of migraine patients, we confirmed the severity of chronic migraine in comparison to episodic patients. CM patients showed more severe anxiety and depression tracts, short sleep time and clear signs of central sensitization, as pericranial tenderness and allodynia [14, 22, 23]. This is confirmative of numerous studies indicating chronic migraine as an invalidating disorder [24].

The inclusion of chronic patients in the study of the circadian rhythm of migraine, is quite difficult considering that headache is often continuous. However, the accurate description of headache characteristics is necessary to individuate the number of migraine attacks, according to recent classifications [7, 8].

Basing on the present population enrolled in our headache centre, the morning onset group included the 10% of patients, less than in previous studies, where the attacks were distributed during the daytime [2–6]. We assigned each patient to a time onset category in an arbitrary way, using the criterion of the 50% of attacks onset, which may be questionable. The different modalities of division in times slots, could explain some differences related to previous studies. In fact we considered the early morning interval from 6 to 12 am, while other studies included the night hours from 0 to 6 [5]. In any case, the preferential time of migraine onset was indicated around the 6 am [4], a time that we included in the morning time slot.

Observing the daily distribution of single migraine attacks, they were disseminated according to a multiphasic circadian rhythm profile, similar to what described by Soriani et al. [25]. Based on this distribution, single attacks did not appear to start very frequently in the early morning.

The type of population selected, headache centre patients vs general population, the modality of data collection, hourly charts vs interview, and the number of patients, seems crucial to explain the divergence with previous studies [2–6]. A comparison between the subjective report of migraine onset and the hourly chart should be useful in the same population. In addition, the geographic, climatic and ethnic characteristics of populations seem another important point, which can profoundly change migraine phenotype. Studies conducted in Italian populations, reported an hourly distribution similar to that emerging by present data, with the exception of night occurrence, which may be age-related [25] (see below).

Our study tried for the first time to establish the circadian profile of migraine attacks in CM patients, and to clarify if chronicity and severity of migraine is associated to specific time of onset categories. No particular circadian rhythm characterized CM patients compared to episodic patients, suggesting that the time of migraine onset could not be a factor associated to the chronic form.

We selected few migraine with aura patients and merged pure migraine with aura and mixed forms in a unique group. The rare presence of night onset attacks may be due to the modality of aura presentation, probably favoured by morning light condition. Few studies dealt with the preferential time of migraine with aura onset. Sunlight could be a trigger for migraine with aura [26]; this could explain the rarity of night onset. In any case, the possibility that migraine aura could start during night without wakening the patient, is an intriguing question. In this case, patients could only report migraine, which occurred after the wake up. A higher number of migraine with aura attacks could have resolved this question.

Our migraine patients reported a higher number of attacks in the night time, in respect to previous studies [2–6]. In addition, considering the time distribution of single migraine attacks and the circadian profile attributed to single patients, most of the night attacks characterized patients with the specific night circadian rhythm. This means that night onset recurs frequently in the same patient, and seems not to occur occasionally. Migraineurs with prevalent night occurrence were older than the others, with the exclusion of morning group. Despite the mean age of the included patients was similar to that of patients reported in other studies [2–6], our MO and CM groups included a high number of over 40 patients. The study by van Oosterhout et al., which included older patients [5], indicated a prevalent migraine occurrence in the time slot 0–6, according to our results. The study of Soriani et al. on circadian rhythms in juvenile migraine [25], reported a low frequency of nocturnal attacks, suggesting that the night occurrence may concur with age-related changes of sleep features. In fact, Alstadhaug et al. [4] described the association between night occurrence of migraine and insomnia. Accordingly, our night-onset patients, showed a reduction of sleep time that confirmed the association previously described [4]. The consideration of other sleep features was beyond the aims of this study, but it would be worthy of further consideration, especially for patients with specific night – onset profile.

In this study, we evaluated for the first time if the characteristics of chronic migraine, as high frequency of attacks and signs of central sensitization, as pericranial tenderness and allodynia, could identify a specific circadian profile. The results are substantially negative in this sense, as frequency of migraine was similar among the different onset time groups, as well as pericranial tenderness and allodynia. As regard to allodynia, the most of migraine patients were classified as allodynic, according to previous studies of our group [14, 15], and other researchers [27–29]. The distribution of allodynic and not allodynic patients was similar among the different circadian categories, so allodynia did not emerge as a peculiar characteristic of a specific circadian profile.

The presence of fatigue characterized a minority of episodic migraine patients, and more than 50% of chronic migraine. A recent study on fatigue in migraine patients, reported an association with poor quality of life, headache intensity, age and insomnia, all symptoms characterizing chronic migraine [14]. In the subgroup of fatigued patients, patients with any time onset prevailed, which could confirm that the presence of fatigue, though relevant especially in chronic patients, is substantially independent from a specific circadian occurrence.

Anxiety and depression tracts were similar in the different circadian categories. Ohayon et al. [30] evaluated the symptoms associated to morning headache in general population and found that anxiety and depression were the most significant associated factors. Our data could not confirm the association between anxiety and depression tracts and the morning migraine. Again, the method of data collection seems crucial for the results. Probably the most of patients have a subjective impression of a circadian prevalence of their attacks. Anxious subjects can emphasize the occurrence of morning headache, because they could dedicate attention to the headache episodes that can influence the quality of the entire day. For these reasons, anxious and depressed persons can give special relevance to morning headaches, so the collection of data from dairies seem important to reduce possible biases.

Quality of life and migraine disability was also similar among the different circadian occurrence times. This is a further confirmation that we could not individuate an onset time of migraine characterizing the more disabled migraine patients.

Age and migraine duration were the only discriminating features among the different circadian profiles. The night-onset migraine is thus a characteristic of older patients with a long migraine history, and could be considered a age related change of migraine phenotype.

The disruption of sleep rhythm linked to aging, could cause night onset migraine in older patients. The role of hypothalamus in migraine attacks onset is going into increasing consideration [31]. The suprachiasmatic nucleus (SCN) of the hypothalamus functions as the master pacemaker to initiate daily synchronization according to the photoperiod. With aging, circadian desynchronization occurs with sleep symptoms and progressive change of sleep-wake behaviour [32, 33]. These age-related modifications could have an important impact on neurodegeneration [33]. The clinical feature of migraine in old patients, needs to be taken into consideration in the global management of diseases linked to aging [34].

### Study limitations

The major limit of the study is the consistency of diaries. We included a high number of data, and we are basing our conclusions on the reliability of what patients reported, which may be questionable. Electronic diaries, included those on smartphones supports [6, 35] seem handy, especially for the notification of onset time; however, also in this case, the reliability of the data is not for granted. We would have also introduced further data in our database, such as the symptomatic treatments use, or the intensity of single migraine attacks and vegetative symptoms. Unfortunately, many diaries did not report these data in a complete way, so we decided not to include them in the final analysis, and to utilize these to confirm the diagnosis [7, 8]. Another limit regards the selection of outpatients from a headache-centre, with a possible bias as compared to general population. Most of the studies on circadian rhythm reported data from selected migraine groups [2, 3, 25], and one study was on a large Dutch people database [5]. Further studies need to be accomplished for the confirmation of present data in general Italian population.

Another potential cause of divergent results from previous studies, was the type of clinical assessment, based on scales for anxiety, depression and sleep largely applied in our hospital, though not of common use in migraine. In any case, the SAS and SDS are validated for detection of psychopathological tracts in general population and useful when the presence of psychiatric diagnosis is an exclusion criteria [17, 18]. The Medical Outcomes Study (MOS) is also of general use in patients with chronic pain [20], and we previously applied it in large headache populations [13, 14].

Another problem was the consistency of data of smaller groups, especially for patients with aura. However, conclusions about the circadian rhythm of migraine considered as a whole, with the inclusion of chronic patients, seem robust, taking also into consideration previous studies [2, 3, 6].

## Conclusions

Based on present data, the most of migraine patients do not report a specific circadian profile of attacks occurrence. High headache frequency, severe disability, psychopathological tracts as well as central sensitization signs, do not match with a specific circadian rhythm of attacks onset. Data of migraine with aura occurrence are presently not definitive, and worthy of further studies. The night onset migraine could be considered an age- related change occurring in the course of the disease. The present results would be considered, in light of population way of selection as well as ethnic and geographic characteristics.

## Additional files

Additional file 1: Headache Diary: Sample of original hourly chart pag.1. (TIF 119 kb)

Additional file 2: Headache Diary: Sample of original hourly chart pag. 2. (TIF 133 kb)

Additional file 3: **Table S1.** Presence and severity of fatigue and allodynia in the migraine subgroups. The results of statistical test are reported as *p* values. **Table S2.** Presence of allodynia in the migraine subgroups and onset time slots. **Table S3.** Presence of fatigue in the onset time slots. **Table S4.** Discriminant analysis Variables ordered basing on absolute dimension of intra function correlation. (DOCX 20 kb)

## Abbreviations

CM: Chronic Migraine
MAF: Multidimensional Assessment of Fatigue
MH: SF 36 Mental Health score
MH: SF36 Physical Health score
MO: Migraine with aura
MO: Migraine without aura
MOS: Medical Outcome Studies
PH: SF36 Physical Health score
SAS: Self-Administered Zung Anxiety Scale
SDS: Self-Administered Zung Depression Scale
SF-36: Short-form 36 Questionnaire (), with the computation of
TTS: Total Tenderness Score
