# Supplementary figures and images for: Circadian rhythms of migraine attacks in episodic and chronic patients: a cross sectional study in a headache center population
Source: BMC Neurol. 2018 Jul 2;18:94. doi: 10.1186/s12883-018-1098-0 (PMC6027564; doi:10.1186/s12883-018-1098-0)

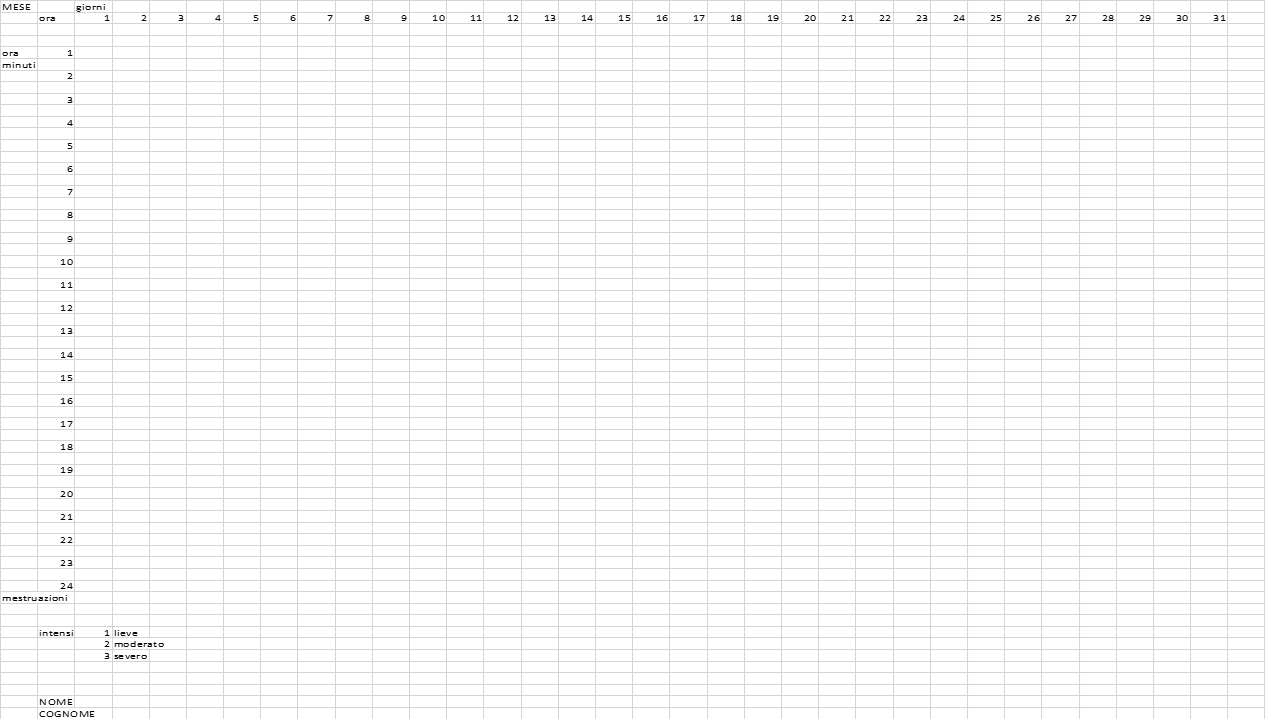

Supplement: Supplementary file 1 — Headache Diary: Sample of original hourly chart pag.1. (TIF 119 kb) [file 12883_2018_1098_MOESM1_ESM.tif]

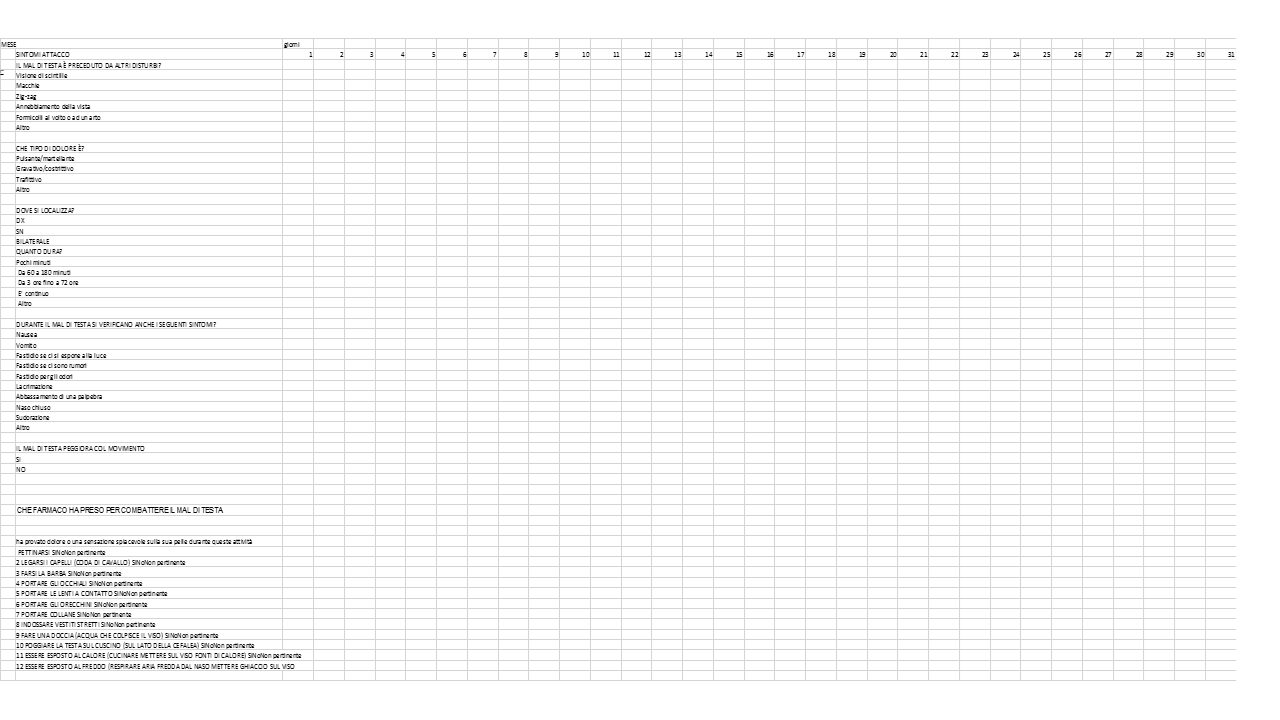

Supplement: Supplementary file 2 — Headache Diary: Sample of original hourly chart pag. 2. (TIF 133 kb) [file 12883_2018_1098_MOESM2_ESM.tif]
