# Supplementary material for: Circadian rhythms of migraine attacks in episodic and chronic patients: a cross sectional study in a headache center population
Source: BMC Neurol. 2018 Jul 2;18:94. doi: 10.1186/s12883-018-1098-0 (PMC6027564; doi:10.1186/s12883-018-1098-0)
Supplement: Supplementary file 3 — Table S1. Presence and severity of fatigue and allodynia in the migraine subgroups. The results of statistical test are reported as p values. Table S2. Presence of allodynia in the migraine subgroups and onset time slots. Table S3. Presence of fatigue in the onset time slots. Table S4. Discriminant analysis Variables ordered basing on absolute dimension of intra function correlation. (DOCX 20 kb) [file 12883_2018_1098_MOESM3_ESM.docx]

|  | MO |  | CM |  | MO+MA |  | total |  |  |
| --- | --- | --- | --- | --- | --- | --- | --- | --- | --- |
| MAF | n | % | N | % | n | % | n | % |  |
| NO | 425 | 78.8 | 100 | 51.5 | 43 | 82.6 | 569 | 72.4 |  |
| YES | 114 | 21.2 | 94 | 48.5 | 9 | 17.4 | 217 | 27.6 | Chi square  0.0001 |
|  | mean | SD | mean | SD | mean | SD |  |  |  |
|  | 45.03 | 21 | 46.19 | 22.04 | 53.33 | 23.6 |  |  | One way Anova  n.s. |
| ALLODYNIA |  |  |  |  |  |  |  |  |  |
| NO | 86 | 16 | 20 | 10.3 | 6 | 11.5 | 112 | 14.2 |  |
| YES | 453 | 84 | 174 | 89.7 | 46 | 88.4 | 674 | 85.8 | Chi square n.s. |
|  | mean | SD | mean | SD | mean | SD | mean | sd |  |
|  | 2.46* | 1.32 | 2.95*^ | 1.35 | 2.2 | 1.37 |  |  | One way Anova 0.00001 |

Table S1 Presence and severity of fatigue and allodynia in the migraine subgroups.. The results of statistical test are reported as p values.

Results of Bonferroni test: *CM vs MO; ° CM vs MO+MA p<0.05

| ALLODYNIA | | | Total |
| --- | --- | --- | --- |
|  |  |  |  |
|  |  |  |  |
| NO | time of migraine onset | MORNING | 14 |
|  |  | ANY TIME | 62 |
|  |  | NIGHT | 25 |
|  |  | AFTERNOON | 9 |
|  |  | EVENING | 2 |
|  | Chi square:10.58 Df 12 p 0.56 | | 112 |
| YES | time of migraine onset | MORNING | 66 |
|  |  | ANY TIME | 387 |
|  |  | NIGHT | 167 |
|  |  | AFTERNOON | 46 |
|  |  | EVENING | 8 |
|  | Chi square: 13.33 DF 12 p 0,329  Total  Chi square 13.56 DF 12 p 0.32 | | 674 |

Table S2 Presence of allodynia in the migraine subgroups and onset time slots.

| FATIGUE | | | Total |
| --- | --- | --- | --- |
|  |  |  |  |
| NO | Time of migraine onset | MORNING | 59 |
|  |  | ANY TIME | 328 |
|  |  | NIGHT | 136 |
|  |  | AFTERNOON | 40 |
|  |  | EVENING | 8 |
|  | Chi square 10,32 p 0.58 | |  |
| YES | Time of migraine onset | MORNING | 21 |
|  |  | ANY TIME | 121 |
|  |  | NIGHT | 56 |
|  |  | AFTERNOON | 15 |
|  |  | EVENING | 2 |
|  | Chi square 23.94 p 0.0001  Total: chi square 13.58 p 0.39 | |  |

Table S3 Presence of fatigue in the onset time slots.

|  | Function | |
| --- | --- | --- |
|  | 1 | 2 |
| Age | ,939^*^ | -,345 |
| Duration | ,796^*^ | ,606 |
| MOS2 | -,281^*^ | ,150 |
| SDS | ,272^*^ | -,130 |
| SAS | ,203^*^ | -,061 |
| FREQUENCY | ,184^*^ | -,041 |
| TTS | ,158^*^ | -,073 |
| ISF | -,116^*^ | ,052 |
| ISM | -,097^*^ | ,065 |
| ALLODYNIA^b^ | ,079^*^ | ,019 |
| Table S4 Discriminant analysis Variables ordered basing on absolute dimension of intra function correlation. | | |
